# Microscopic Origin of Polarity‐Dependent V_TH_ Shift in Amorphous Chalcogenides for 3D Self‐Selecting Memory

**DOI:** 10.1002/advs.202408028

**Published:** 2024-10-09

**Authors:** Ha‐Jun Sung, Minwoo Choi, Zhe Wu, Hwasung Chae, Sung Heo, Youngjae Kang, Bonwon Koo, Jong‐Bong Park, Wooyoung Yang, Yongyoung Park, Yongnam Ham, Kiyeon Yang, Chang Seung Lee

**Affiliations:** ^1^ Thin Film Technical Unit Samsung Advanced Institute of Technology Samsung Electronics Suwon‐si 16677 South Korea; ^2^ Advanced Process Development Team Semiconductor R&D Center Samsung Electronics Hwaseong‐si 18448 South Korea; ^3^ Analytical Engineering Group Samsung Advanced Institute of Technology Samsung Electronics Suwon‐si 16677 South Korea

**Keywords:** amorphous chalcogenides, self‐selecting memory, ovonic threshold switching

## Abstract

Ovonic threshold switching (OTS) selectors based on amorphous chalcogenides can revolutionize 3D memory technology owing to their self‐selecting memory (SSM) behavior. However, the complex mechanism governing the memory writing operation limits compositional and device optimization. This study investigates the mechanism behind the polarity‐dependent threshold voltage shift (ΔV_TH_) through theoretical and experimental analyses. By examining the physical principles of threshold switching and conducting defect state analysis, the ΔV_TH_ as a memory window is confirmed to be attributed to the dynamics of charged defects and their gradient near electrodes, influenced by the nonuniform electric field after threshold switching. This study provides critical insights into the operational mechanism of OTS‐based SSM, known as selector‐only memory, highlighting its advantages for developing high‐density, low‐cost, and energy‐efficient memory technologies in the artificial intelligence era.

## Graphical abstract

This study explores the mechanism behind polarity‐dependent threshold voltage shifts (ΔV_TH_) in ovonic threshold switching selectors using amorphous chalcogenides. Through theoretical and experimental analyses, it reveals that the ΔV_TH_ originates from charged Se and cation dimer defect redistribution near electrodes, influenced by nonuniform electric fields, providing insights for optimizing self‐selecting memory devices in 3D memory technology.

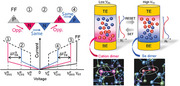

## 1 Introduction

Chalcogenide materials, non‐oxide compounds alloyed with group 16 elements, such as S, Se, and Te, and often alloyed with group 15 (As, Sb, and Bi) or group 14 (Si, Ge, Sn, and Pb) elements, have gained significant interest owing to their unique properties for energy and electronic applications. These materials have been integral in various nonvolatile memory technologies, such as phase‐change memory (PCM) and conductive‐bridging random‐access memory (CBRAM) for neuromorphic electronics.^[^ ^1^, ^2^, ^3^, ^4^ ^]^ The notable electronic nonlinearities of the ovonic threshold switching (OTS) mechanism observed in some chalcogenide glasses under an electrical field make them prime materials for innovative selector elements in 3Dcross‐point (3DXP) array, suppressing sneak currents.^[^ ^5^ ^]^ Combining OTS and PCM in a one‐selector one‐resistor (1S1R) configuration can achieve 4F^2^ footprint, crucial for high‐density stacking, a key aspect of scaling opportunities.^[^ ^6^ ^]^

The 3DXP array architecture composed of PCM and OTS is highly promising for storage‐class memory applications in the memory hierarchy, as it meets the requirements of low cost and high density.^[^ ^7^, ^8^ ^]^ However, PCM, operating by switching between amorphous (RESET) and crystalline (SET) states via Joule heating, faces challenges in energy efficiency and operational speed owing to its high current requirements. In addition, the 1S1R structure increases process complexity and results in a poor aspect ratio, leading to higher cost/bit with over four stacking layers. To address these issues, recent advancements have introduced the concept of self‐selecting memory (SSM), which uses a single chalcogenide material and utilizes polarity‐induced threshold voltage (V_TH_) shifts in OTS.^[^ ^9^ ^]^ For instance, Micron has announced the development of a 256 Gb memory with a 4‐deck array structure, achieving a one‐selector zero‐resistor (1S0R) configuration.^[^ ^10^ ^]^ This design is superior to that of traditional PCM in terms of operational speed, current usage, and stability because its operation relies on voltage‐induced switching rather than repetitive amorphous–crystalline phase changes.

The concept of OTS‐based selector‐only memory (SOM) has emerged as a pivotal innovation in‐memory technology, demonstrating superior performance and significant simplification in cell structure, and offering substantial potential for scaling. Despite these advancements, the operational principles of SOM, particularly the mechanisms driving the V_TH_ shift in response to polarity, remain a subject of ongoing research and exploration.^[^ ^11^ ^]^ Unlike PCM, SOM exhibits reversible movement between low V_TH_ (SET) and high V_TH_ (RESET) states while maintaining an amorphous structure, influenced by polarity. The mechanism behind this polarity effect is believed to be influenced by the material and operating current. One hypothesis suggests that elemental segregation could contribute to the V_TH_ shift.^[^ ^12^, ^13^ ^]^ However, this atomic movement has not been observed under low‐operating‐current conditions, necessitating further investigation into the underlying processes governing SOM behavior. Other hypotheses attribute the polarity effect to electronic origins, such as asymmetric hole‐trapping phenomena in PCM and anisotropic structural units by delocalized defects.^[^ ^9^, ^14^, ^15^, ^16^ ^]^ Although previous research has partly discussed the polarity effect, a precise understanding of its microscopic origin in OTS materials and the mechanism behind remains lacking. Mechanistic understanding of the polarity effect in OTS is crucial for developing efficient SOM, considering its key role in the material design of SOM as a next‐generation 3D device.

This study proposes a mechanism for the polarity‐dependent V_TH_ shift observed in OTS materials, using the amorphous Ge‐As‐Se (a‐GAS) series as a model system, which offers low leakage current and high thermal stability for 3D stackable cross‐point memory applications.^[^ ^17^, ^18^ ^]^ We identified that the writing pulse with different polarity causes the redistribution of charged defects, linked to two distinct Poole–Frenkel (PF) mechanisms. The microscopic origin of these charged defects, specifically dimer defects, is further supported by density functional theory (DFT) simulations and deep level transient spectroscopy (DLTS) results. Additionally, we suggest that charged defects are redistributed near the electrodes by a non‐uniform electric field under different polarity writing pulses. Furthermore, we investigated the geometric and compositional effects on the memory window, and found a trade‐off between the memory window and device reliability.

In the next section, we introduce the polarity‐dependent V_TH_ shift based on different PF conduction in SOM device. Section 3 focuses on the microscopic origin of defect states that act as a PF emission site. Section 4 provides a discussion on the operation mechanism of SOM device, including operation pulse scheme and other possible mechanisms. Finally, Section 5 focuses on the device structure dependency based on the proposed model, along with adiscussion on its performance.

## 2 Polarity‐Dependent V_TH_ Shift

**Figure 1:**
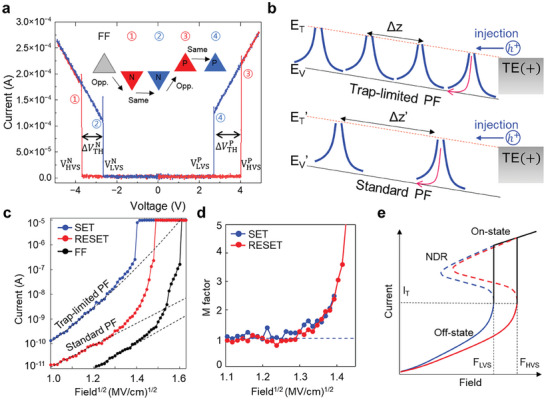
Electrical characteristics of SOM devices and polarity‐dependent V_TH_ shift phenomena. a) Bipolar pulses and typical current–voltage curve of the OTS device, indicating the polarity effect. A sequence of bipolar pulses in same or opposite polarity is applied after first‐firing (FF) operation. NNPP pulse‐train has paired positive and negative triangular pulses (inset). b) Schematic of the thermal emission of trapped carrier during positive read operations corresponding to small and large trap distances (Δz). The trap‐limited PF conduction for a small Δz (high trap density) shows a linear decrease in the potential barrier with the applied field (Δϕ∝|*V_a_* |). For a large Δ*z* (low trap density), where the carrier emission considers only isolated traps, the barrier lowering is proportional to the square root of the field $\left(\Delta \phi \propto |\sqrt{V_{a}}|\right)$. c) Measured current for a 16 nm thick device as a function of the square root of the field in SET(LVS), RESET(HVS), and FF states, highlighting the trap‐limited PF and standard PF conduction models (dashed line). At higher fields, just before the switching field, the current exhibits a superexponential increase over the PF trend in both cases. d) Extraction of avalanche multiplication factor M as a function of the electric field. e) Schematic diagram of *I–V* characteristics with LVS and HVS in a typical SOM device. The S‐shaped NDR characteristic is illustrated under current‐controlled operation (dashed line) and threshold switching under voltage‐controlled mode (solid black line).

**Figure** **1**a shows the schematic illustration of polarity‐dependent *I–V* curve characteristics during the measurement sequence of chalcogenide memory. In the threshold switching process, the devices initially exhibit high resistance in the low‐bias region, referred to as the OFF state. As the applied voltage gradually increases, the devices abruptly switch to the ON state. In this state, the OTS thin films transition to a low‐resistance state at a specific voltage known as the threshold voltage. A forming process is necessary during the first‐firing pulse, which requires a larger voltage to switch (not shown here). The V_TH_ state of the OTS is determined by whether the polarity of a sequence of pairs of pulses is the same or opposite. We present the NNPP (double negative, double positive) pulse‐train measurement to explain the polarity‐dependent V_TH_ shift. This measurement consists of pairs of negative and positive triangular pulses (inset in Figure 1a). Before conducting the NNPP measurement, it is necessary to apply an initializing pulse to switch in the positive direction. In each pair, the first pulse (① and ③, red curve) has an opposite polarity and switches to the high V_TH_ state (V_HVS_), while the second pulse (② and ④, blue curve) has the same polarity and results in a low V_TH_ state (V_LVS_). Consequently, a polarity‐driven V_TH_ shift is observed with both positive and negative pulses $\left(\Delta V_{\mathrm{TH}}^{P,N}=|V_{\mathrm{HVS}}^{P,N}\left|\;-\right|V_{\mathrm{LVS}}^{P,N}|\right)$. In our device, the threshold voltage for the first firing is ≈5 V, while the operating range for V_LVS_ and V_HVS_ is ≈3 and 4 V in positive direction for 16 nm thick OTS devices, respectively.

The electrical conduction in the subthreshold region of OTS is believed to be governed by thermally activated PF transport over the potential barrier by majority holes at low field region, expressed as $I_{\mathrm{PF}}\propto \exp\left(-\phi /\mathrm{kT}\right)$. The change in defect concentration by external stimulus can strongly affect the switching properties and the conductivity of the OTS device.^[^ ^19^ ^]^ Figure 1b shows the possible PF conduction mechanism in OTS, which depends on trap density. For a large trap density, trapped carriers move along the direction of the electrostatic force with a potential barrier ϕ defined as $\phi =E_{V}-E_{T}-q\frac{\Delta z}{2u_{a}}V_{a}$ for trap‐limited PF emission, while the emission from an isolated trap to the valence band exhibits a barrier expressed as 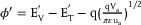 for standard PF emission.^[^ ^20^ ^]^ In these equations, *E*_V_ is the valance band edge, *E*_T_ is the trap level, Δ*z* is the intertrap distance, *u*_a_ is the film thickness, *V_a_* is the applied voltage, *ε* is the dielectric constant, and *q* is the elemental charge.

Here, we suggest the polarity‐dependent control of PF conduction mechanism. Figure 1c shows the measured *I–V* curves of an OTS device with *a*‐GAS in the first firing, SET, and RESET states in positive direction. After a write pulse applied (negative polarity pulse for RESET, positive polarity pulse for SET states), V_TH_ of the cell changes ($V_{\mathrm{HVS}}^{P}$ > $V_{\mathrm{LVS}}^{P}$). To measure the conduction and switching characteristics, the OTS device was connected in metal to form a metal/OTS/metal structure, allowing a tight control of the maximum compliance current I_C_ = 10 uA after threshold switching. For the SET state, the off‐conduction state under a low applied voltage is well fitted by a trap‐limited PF model with  *E*_V_ − *E*_T_ ≈0.49 eV and Δ*z* ≈4.6 nm (*N*_T_ ≈1.1  ×  10^19^ cm^−3^). The off‐conduction slope of the RESET and FF states appears similar to that of standard PF conduction with *ε* ≈6.7, a reasonable value for *a*‐GAS.^[^ ^21^ ^]^ Considering the OFF state in the first firing as trap‐limited PF conduction rather than standard, the slope of the *I–V* curve is fitted with an intertrap distance Δ*z* = 3.4 nm (*N*_T_ ≈2.5  ×  10^19^ cm^−3^) under 1.4 (MV/cm)^1/2^. This indicates that the trap density decreased by ≈2.3 times after the first firing. However, we confirmed that the trap density of OTS increases in the first firing process via photonic *I–V* trap measurement (Figure S1, Supporting Information), indicating that the off conduction in the RESET and first firing states is not trap‐limited PF but standard PF.

The off‐conduction current in the RESET state is significantly lower than that in the SET state owing to differences in the barrier‐lowering coefficient with the electric field. However, both states exhibit a superexponential increase in current at higher fields, attributed to avalanche carrier multiplication resulting from impact ionization (II). Figure 1d illustrates the multiplication factor (M) in the SET and RESET states, defined as the ratio of the bias current I to the premultiplication current initiated by PF emission, M = I/I_PF_. Although the OFF‐state conduction mechanism shows two different PF transports corresponding to a high or low trap concentration, the M trend is similar in both states over a square root of electric field of 1.3 (MV cm)^−1/2^. The film thickness (*u_a_*) influences the M, expressed as M = exp(α  ×  *u_a_*). The variation in the switching field with increasing thickness is discussed in relation to the device structure in the last section. The field‐dependence of the II coefficient (α) can be extracted from the measured M (Figure S2, Supporting Information). The threshold energy of the material significantly influences the α, resulting in little to no influence based on polarity. The lucky‐drift model^[^ ^22^ ^]^ is possible in materials such as amorphous chalcogenides, where a significant number of in‐gap states exist causing the Fermi level to be pinned at the midgap. In this case, the threshold energy (E_I_) is approximately E_g_/2. Most recently, Fantini et al. reported that the OTS mechanism is triggered by electron–hole cooperation by bipolar II to explain the S‐shaped negative differential resistance (NDR), as shown in Figure 1e.^[^ ^23^ ^]^ When the current surpasses the critical threshold, the voltage decreases, leading to the S‐shaped NDR that underlies the threshold switching mechanism. A higher voltage is necessary for threshold switching owing to the slower increase in current with respect to the field in the OFF state of the HVS compared with the LVS.

## 3 Microscopic Origin of Memory Window

Understanding the defect structures of amorphous chalcogenides is necessary to explain the defect redistribution during SET and RESET operations. Assuming that only point‐like defects are present in amorphous materials is logical because extended defects require long‐range atomic correlations, which are lacking in the non‐crystalline state. The types of point defects that create gap states in amorphous chalcogenides include coordination defects and dimer‐type wrong bond defects. Singly charged coordination‐defect (valence‐alternation) pairs hold particular importance when considering photoinduced structural changes.^[^ ^24^, ^25^ ^]^ The well‐known valence‐alternation pairs (VAPs) are $C_{1}^{-}$‐$C_{3}^{+}$, $P_{4}^{-}$‐$C_{3}^{+}$, and $P_{4}^{+}$‐$C_{1}^{-}$, where *C*_n_ and *P*_n_ denotes for an n‐fold coordinated chalcogen and pnictogen (e.g., Se, As) atom. However, it is important to note that this process cannot occur for group‐IV elements (e.g., Ge) because they cannot be fivefold coordinated. Meanwhile, dimer‐type defects (e.g., Se─Se, Ge─Ge, As─As, and Ge─As bonds in a‐GAS) cause major source of gap states, which play a crucial role in OTS behavior.^[^ ^26^, ^27^ ^]^ Hereafter, chalcogen and cation wrong bonds are referred to as Se and cation dimers, respectively.

Dimer‐type defects exist more frequently in compound amorphous chalcogenides than coordination defects, such as VAPs, owing to their lower defect creation energy. In binary amorphous chalcogenides, such as As_2_Se_3_, dimer formation is physically governed by the stronger hetero‐bonds (As─Se), having partial ionic characteristics, compared with the average strength of various dimer bonds (As─As or Se─Se). Simple dimer defects exhibit a dimer creation energy (ΔE) of ≈0.18 eV for As_2_Se_3_ and 0.38 eV for GeSe_2_. These values were derived from a semiempirical formula for bond dissociation energies, expressed as ΔE =(*x_A_x_B_*)^2^[*e*V], where *X_A_* and *X_B_* denote the electronegativity of each species. Assuming that defects are frozen at the glass‐transition temperature (T_g_), the density of each defect can be expressed as n_0_  ×  exp(−ΔE/kT_g_), where n_0_ is the approximated density of the atomic sites. The estimated concentration of dimer structures emerging from the glass at T_g_ is ≈10^20^–10^21^ cm^−3^. By contrast, the creation energy of VAPs is larger (≈0.8 eV in *a*‐Se) and less thermally stable.^[^ ^24^ ^]^ Paramagnetic centers in GeSe_2_ (5  ×  10^15^ cm^−3^) and As_2_Se_3_ (10^17^ cm^−3^), related to VAPs in amorphous chalcogenides, have been detected through optically induced electron‐spin resonance signals.^[^ ^28^ ^]^ Furthermore, because the charge balance of coordination defects is easily compensated by dimer defects in amorphous chalcogenides, the concentration of VAPs is not expected to be highly stoichiometry‐dependent.

**Figure 2:**
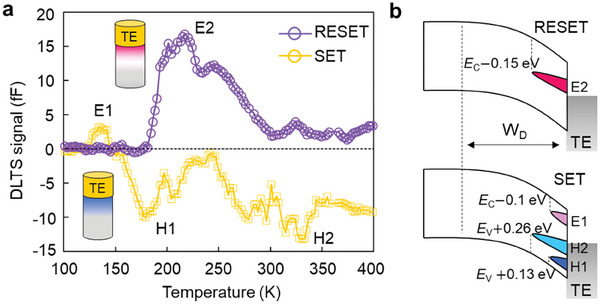
Defect characterization under electrical operation. a) DLTS signal of the SET and RESET states in the OTS device at a pulse voltage of 0.5 V. The inset shows the corresponding defect distribution in the device, with red and blue representing the electron and hole traps, respectively. b) Schematic illustration showing the relative energy positions (E1, E2, H1, and H2) and trap concentrations indicated by the intensity of the four detected trap states in the bandgap. In the DLTS analysis, the detected traps are in the depletion region of the OTS films, W_D_.

DLTS measurements were performed to gain insights into defect modulation during electrical operation. **Figure** **2**a shows the DLTS spectra of electrically operated OTS devices at a pulse height of 0.5 V after different writing polarities. The positive peaks in the DLTS spectra represent electron carrier traps, while the negative peaks indicate hole carrier traps. **Table** **1** summarizes the defect information of the SET and RESET states, where E_T_ and N_T_ are the trap energy level and trap density, respectively. DLTS investigates defects present in the depletion width of the Ge─As─Se system (Figure 2b). The SET state exhibited one electron and two hole traps (E1, H1, and H2), with energy levels of *E*_C_− 0.1, *E*_V_+ 0.13, and *E*_V_+ 0.26 eV, respectively. H2 defects predominantly appeared in the SET state. By contrast, after RESET operations, the defect profiles near the interface were dominated by E2 with an energy level of *E*_C_− 0.15eV. This redistribution of charged defects near the interface is a reversible process. Traps H1 and E1 can be assigned to $C_{1}^{-}$ and $C_{3}^{+}$ of VAP defects confirmed by DFT simulations. The energy levels of VAP are calculated to be *E*_C_− 0.13 eV and *E*_V_+ 0.12 eV for *C*_3_ and *C*_1_, respectively. To estimate the VAP defect level, we considered a pure Se amorphous model containing VAPs (Figure S3, Supporting Information).

**Table 1:** Measured trap concentration (N_T_) and trap level (E_T_).

		N_T_ [cm^−3^]	E_T_ [eV]
SET	E_1_	2 × 10^16^	E_C_− 0.1
	H_1_	7 × 10^16^	E_V_ + 0.13
	H_2_	1 × 10^17^	E_V_+ 0.26
RESET	E_2_	1.3 × 10^17^	E_C_− 0.15

**Figure 3:**
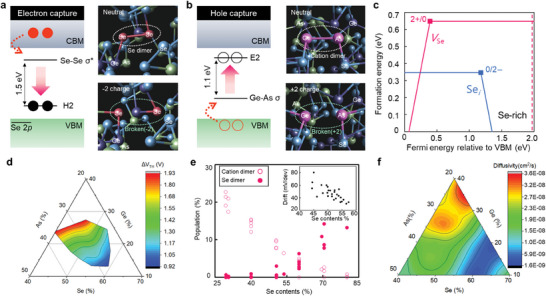
Microscopic origin of defect states and Ge─As─Se ternary diagram. a) The Se‐Se σ* state is lowered to the valence band maximum as the bond length of the dimer extends upon capturing two electrons. The atomic structures of the Se dimer in the neutral and ‐2 charge states (right panel). b) The Ge─As σ state moves upward to the conduction band minimum upon capturing two holes. The atomic structures of the cation dimer in the neutral and +2 charge states (right panel). The red and pink spheres denote the Se and cation dimers, respectively. c) Average formation energy of the Se*_i_* and *V*_Se_ defects under Se‐rich conditions. The thermodynamical transition levels of 2+/0 and 0/2‐ are indicated by filled square. The red dashed line denotes the bandgap of 1.97 eV for *a*‐GAS (Ge_20_As_20_Se_60_). d) Ternary diagram of the measured memory window ΔV_TH_. e) Calculated population of Se and cation dimers in an amorphous structure as a function of Se composition. The inset shows the measured drift coefficient in the mushroom‐type OTS device. f) Diffusivity map obtained from DFT simulation. The mean squared displacement (MSD) was calculated to extract the diffusion coefficient. The MSD was obtained from 100 ps AIMD simulations at 300 K.

Next, to examine the origin of E2 and H2 defects, DFT calculations were performed for dimer‐type defects in the a‐GAS system. Under thermal equilibrium conditions, high‐Se compositions of *a*‐GAS predominantly form Se dimers, whereas cation dimers are easily formed in low‐Se compositions. Although the dimer defects are neutral in charge in amorphous chalcogenides, double ionization is induced by the carrier‐capture process during OTS switching (See detailed capture process in Section S1 and Figure S4, Supporting Information). In our previous experiments, we confirmed that the binding energy of Se 3*p* was shifted under a high applied field measured by operando XPS spectra.^[^ ^29^ ^]^ This observation provides insight that Se‐related defects can be ionized during the threshold switching. When a large concentration of electrons is injected from the electrode by applying a sufficient electric field for threshold switching in OTS, they can be captured by the Se─Se anti‐bonding state, leading to outward relaxations and eventual bond breaking (**Figure** **3**a). The defect level of the broken Se dimer in the double‐charged state is ≈0.3 eV higher than the valence band maximum (VBM), consistent with the H2 state in the DLTS results (Figure S5, Supporting Information). The energy disparity between the two defect levels contributes to the overall energy gain. Furthermore, as the cation dimers capture two holes, the bonding state is emptied and transitions to a nonbonding defect state with bond‐breaking relaxation, located ≈0.2 eV below the conduction band edge, corresponding to the E2 defect (Figure 3b).

Mobile ionic species in solids are linked to point defects, such as interstitial or vacancy defects. Se dimer defects are categorized as Se‐interstitial (Se*_i_*) defects, exhibiting deep subgap states formed by the σ interaction between chalcogens. By contrast, cation dimer defects act as electron traps, similar to Se vacancy (*V*_Se_) defects. In the neutral state, an Se interstitial defect forms an Se dimer with one of the host Se atoms in the most stable configuration. Furthermore, the distribution of charged defects near the interface significantly changes with the position of the Fermi level shift under SET and RESET pulses. The formation energy of Se*_i_* and *V*_Se_ strongly depends on their local geometry surrounded by Se or cation atoms, denoted as Se‐rich or Se‐poor conditions, respectively (details in Experimental Section). The results of DFT calculations indicate the average formation energies of ${\mathrm{Se}}_{i}^{0}$ and ${\mathrm{Se}}_{i}^{2-}$, varying with the Fermi energy. These were analyzed alongside those of an ionized *V*_Se_ defect under Se‐rich conditions (Figure 3c). Se*_i_* defects dominated under Se‐rich conditions, while *V*_Se_ defects stabilized under Se‐poor conditions (Figure S6, Supporting Information). For *V*_Se_ and Se*_i_*, the thermodynamic transition levels were estimated to be 0.4 eV above the VBM and 0.8 eV below the CBM, respectively. Notably, the single charge state of *V*_Se_ and Se*_i_* was found to be metastable, regardless of the Fermi level position. Thus, both native defects exhibited a negative‐U behavior and acted as a double acceptor or donor. In the negative‐U model, the two identical carriers paring at the same defect center overcame the Coulomb repulsion,^[^ ^30^ ^]^ leading to a net effective attractive interaction between the carriers coupled with significant lattice relaxation similar to the bond breaking in Se and cation dimer structures. Under a positive write pulse, ${\mathrm{Se}}_{i}^{2-}$ defects predominated at the top electrode (TE) side when the Fermi level approached the conduction band minimum (CBM). By contrast, as the Fermi level decreased under a RESET pulse, the neutral Se dimer configuration could be recovered without an energy barrier while the Se vacancy was ionized. The broken‐dimer structure easily relaxed to the dimer state upon neutralization. Therefore, PF conduction mechanism in OFF‐state can be significantly altered by the opposite polarity pulse, driven by charged‐defect generation and annihilation near the electrodes (Figure 2b).

To investigate the changes in dimer configuration and electrical properties based on the composition, amorphous Ge─As─Se was deposited using the co‐sputtering method. Detailed information is provided in Table S1 (Supporting Information). Figure 3d shows that the ΔV_TH_ varies with Se composition, ranging from ΔV_TH_ ≈0.9 V in Se 57% to ΔV_TH_ ≈1.9 V in Se 39%. In low‐Se‐composition region, the increase in cation dimers may influence the ΔV_TH_ (Figure 3e). Notably, ΔV_TH_ was found to be related to V_TH_ drift over time, occurring during the structural stabilization process in the amorphous structure (inset of Figure 3e; Figure S7, Supporting Information). Nudged elastic band (NEB) barrier simulations estimated a low energy barrier of 48 meV to break the bond for dimer and transition to stable heteropolar bonds, such as Ge─Se or As─Se (Figure S8, Supporting Information). The averaged diffusivity of elements extracted from the mean displacement distance in molecular dynamics simulations increased in the low‐Se region (Figure 3f). The redistribution of charge defects caused by the RESET operation measured in DLTS appears to occur more effectively in compositions with higher diffusivity. It is important to note that chalcogenides with significant V_TH_ drift exhibit reduced memory reliability over time. Therefore, despite offering a larger memory window, the trade‐off associated with their use should be considered.

## 4 SOM Operation Mechanism

**Figure 4:**
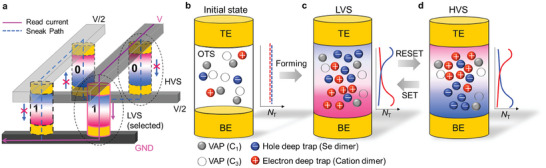
Crossbar memory array of an SSM cell based on amorphous chalcogenides. a) Schematic of the SSM within the crossbar memory array framework, distinct from the conventional 1S1R. Schematic illustration of OTS in the b) initial state, c) low V_TH_ state (LVS) of the selected cell, and d) high V_TH_ state (HVS) of the unselected cell. The filled and open gray circle represent VAP as C_1_ and C_3_, respectively. The red and blue spheres represent electron and hole deep traps originating from cation and Se dimers, respectively (see main text). The charged defects in the OTS cell are uniformly distributed in the initial state, while the distribution of charged defects is reversely altered by SET and RESET operations. The red and blue solid lines on the right side of each cell structure denote the population of electron and hole traps, respectively.

In a 3DXP architecture, the polarity‐dependent V_TH_ shift of the OTS can be leveraged as a memory window for simultaneously performing the selecting and storing operations by applying an SSM. As illustrated in **Figure** **4**a, this is feasible owing to the low leakage characteristics of the OTS, effectively blocking all possible sneak current paths regardless of the “0” or “1” (RESET or SET) state. In this device, the storage element based on resistance memory is completely removed, and the threshold voltage of the OTS selector is used for storing information, thus termed SOM. The read operation of SOM device is nondestructive (detailed operation scheme in Section S2 and Figure S9, Supporting Information). When a read voltage between V_LVS_ and V_HVS_ is applied to a selected cell, if the state was SET, it activates, whereas if it was RESET, threshold switching does not occur, allowing for the distinction between the “0” and “1” states with at least 10^4^ high selectivity (I_on_/I_off_). However, repeated read pulses under sub‐V_TH_ stress (> 10^6^ times) can disturb a stored “0” state (high V_TH_ state).^[^ ^15^, ^31^ ^]^ Moreover, the memory window narrows and eventually fully closes under a sufficiently prolonged sub‐threshold field (> 10 s).^[^ ^16^ ^]^ In the following paragraph, we propose a SOM operation mechanism based on the polarity‐dependent charged defect distribution, revealed through DFT calculations and DLTS analysis. This distribution of charged defects can be disturbed by prolonged sub‐V_TH_ stress due to electromigration force.

In the initial state, point defects, such as dimer defects and VAPs, are randomly distributed in amorphous chalcogenides (Figure 4b). The sharp decrease in V_TH_ resulting from the forming process in OTS appears to be caused by an increase in carrier emission sites along the conduction path in the off‐conduction state, considered as trap‐limited PF emission mentioned above (Figure 4c). Dimer‐type defects can be activated by the forming process, and they comprise an off‐conduction filament promoting a charged‐defect gradient locally around the filament. The ionized Se dimer defects, acting as hole traps, can diffuse toward the top interface, whereas the ionized cation dimer moves toward the opposite interface under LVS. For a positive read scheme, when hole carriers are injected from the TE in the read operation, the potential barrier for thermal emission decreases linearly with the applied field owing to the trap‐limited PF effect with a large concentration of Coulombic traps, leading to a low V_TH_. However, the distribution of these charged defects is altered by the opposite polarity pulse, evidenced by DLTS analysis presented above. When a negative writing pulse is applied, the hole emission site is repulsed from the TE, increasing the Δ*z* in the subsequent read operation (positive read) (Figure 4d). Thus, the OFF‐state conduction mechanism changes into standard PF with carrier emission from an isolated hole trap, with potential barrier lowering proportional to the square root of the applied field.

Furthermore, the read operation can also be performed in negative direction (Figure S9, Supporting Information). For negative read scheme, the cell written with a negative pulse has ionized Se dimers (hole trap sites) concentrated at the BE, so in the next read, the hole carriers injected from the BE undergo trap‐limited PF conduction. This is the same situation that occurs in a cell written with a positive pulse for a positive read scheme (Figure 4c). The operation scheme of the SOM device depends on whether the polarity of the previous write pulse is the same or opposite to the read direction, which in turn determines the V_TH_ value. For example, negative writing state is represented by HVS for a positive read and LVS for a negative read. However, in practical cross‐point memory operations, only one read polarity is required.

Literature suggests that chalcogenide materials may experience segregation upon the application of an electric field, with different atomic species migrating toward the opposite electrodes, depending on their relative electronegativity.^[^ ^11^, ^12^, ^13^ ^]^ Hong et al. demonstrated memory function reliant on the polarity effect and proposed elemental segregation as a potential mechanism.^[^ ^12^ ^]^ This may explain the difference in the V_TH_ and I_off_ properties of the OTS under different pulse polarities owing to the change in local composition. However, significant atomic migration typically occurs under high‐operating‐current conditions, I_op_ > few mA.^[^ ^13^ ^]^ PCM based on Ge─Sb─Te increases its temperature to the melting point owing to Joule heating during operation, suggesting a possible migration effect. In OTS, migration occurs only with high current, long pulse time, or in endurance‐failed samples, even when it switches to the ON‐state.^[^ ^32^ ^]^

Ravsher et al. have been recent efforts to explain the polarity effect of OTS based on anisotropic structural units (ASUs).^[^ ^16^ ^]^ The main assumption of the ASU model is the presence of spatially extended defects with anisotropic nature, making it sensitive to polarity. The nature of the presumed anisotropic defects has not been provided yet. Meanwhile, the Coulomb attraction between $C_{3}^{+}$ and $C_{1}^{-}$ makes it likely that a pair of these defects is in close proximity, so called intimate valance alternation pairs (IVAPs) in amorphous chalcogenides. ASUs may be linked to certain atomic environments with inherent asymmetry, such as IVAPs. DLTS analysis confirmed that E2, H2 (doubly charged broken dimers) states are more sensitively changed by polarity than E1, H1 (singly charged VAPs) states. However, this result does not mean that the dimer‐type defects are candidates for ASUs. Since dimer defects are randomly distributed in as‐deposited amorphous state, there is no physical reason for them to be in close proximity.

## 5 SOM Device Properties Modulation

This section focuses on the characteristic features of the electrical properties in the proposed OTS‐based SSM device. The endurance of OTS devices, derived from their switch behavior, supplants the recurring phase‐transition found in PCM, significantly enhancing their endurance.^[^ ^10^ ^]^ Moreover, leveraging the threshold voltage of the OTS device rather than the operating current of the PCM eliminates the need to melt the chalcogenide material, significantly reducing energy consumption. Under repeated writing pulses to the SSM device, it was confirmed that the memory window is maintained up to 100M cycles. (Figure S10, Supporting Information) This failure mechanism is attributed to atomic migration caused by repeated bias stress, similar to what occurs with selector device. To reduce this phenomenon, recent studies have been published on controlling the spike current caused by RESET/SET writing pulses.^[^ ^32^ ^]^ Moreover, process technologies are being developed to reduce failure points caused by damage to the side walls of OTS pillars during the integration process.

**Figure 5:**
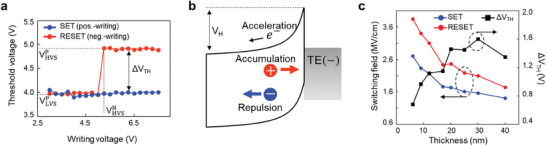
*V–V* curve in SOM device and thickness‐dependent switching field. a) Writing voltage and threshold voltage (*V–V*) curve in the proposed pillar‐type device (Experimental Section). The blue and red points denote the threshold voltage for writing pulses in the same and opposite directions, respectively, with respect to the read direction. $V_{\mathrm{LVS}}^{P}$, $V_{\mathrm{HVS}}^{P}$, $V_{\mathrm{HVS}}^{N}$, and ΔV_TH_ are indicated in Figure 1a. b) Nonuniform electric field with high field near the electrode after threshold switching in the OTS film under reverse writing operation. Positively charged defects accumulated to the TE side, whereas negatively charged defects are repulsed. c) Thickness‐dependent threshold switching field and ΔV_TH_. Because the difference between the switching field of the SET and RESET states decreases with thickness from 6 to 40 nm, ΔV_TH_ becomes saturated and then decreases at higher thicknesses. This thickness‐dependent switching field reduction is also observed in the pillar‐type device.

**Figure** **5**a shows the remarkable *V–V* curve of OTS in polarity‐dependent V_TH_ shift trends. As the magnitude of the negative writing voltage is increased, reading the V_TH_ in the positive direction suddenly creates a memory window (ΔV_TH_) at a specific writing voltage corresponding to $V_{\mathrm{HVS}}^{N}$. Interestingly, threshold switching in the negative direction only occurs above $V_{\mathrm{HVS}}^{N}$, indicating a relationship between the memory window and the threshold switching mechanism in the opposite polarity direction. Thus, the operating speed was identical to the threshold switching speed of the OTS device, approximately subnanosecond level in the proposed device (Figure S11, Supporting Information). The speed achieved with a‐GAS, measured at 720 ps, is already quite fast. Figure S12 (Supporting Information) indicate that the operation speed was evaluated through three repeated measurements. It can be observed that the memory window is no longer found at pulse durations below 0.7 ns. However, the operational speed could be further enhanced by optimizing the device structure or by incorporating dopants that can increase the diffusivity of charged defects related to the operation mechanism. Figure S13a–c (Supporting Information) shows the related device yield and the uniformity of the threshold voltage based on DC *I–V* characteristics. A total of 135 devices were confirmed within an operating voltage range of ±0.5V. In particular, the uniformity between devices in the four sections of the wafer, which were divided using the combinatorial methodology, was also found to be excellent, as confirmed by the wafer mapping data in Figure S13d (Supporting Information). Additionally, we measured the temperature dependence of the operation, and as the temperature increased, both SET V_TH_ and RESET V_TH_ slightly decreased, resulting in no significant change in the memory window (Figure S14, Supporting Information).

Threshold switching in the opposite direction is necessary for RESET writing by creating a large electric field near the cathode interface. It should be noted that the increase in conductivity resulting from avalanche multiplication may not explain the NDR state and threshold switching effect. The nonuniform profile of the electric field is necessary to explain the NDR behavior, as shown in Figure 5b. The voltage drop across the OTS film in the highly conductive state remained constant, corresponding to the holding voltage (V_H_). Therefore, the memory window did not increase, even under high writing voltages. The field distribution near the electrode induced the redistribution of the charged trap profile by the high electromagnetic force on the ionic defect. The field decreased close to the bulk region because the large current of the ON state could be sustained by the large concentration of high‐mobility secondary carriers without requiring a high electric field.^[^ ^19^ ^]^ The electric field distribution varied nonuniformly across the amorphous layer owing to the finite distance dead space required for carriers to gain the ionization threshold energy, becoming more crucial in thinner structures. Meanwhile, Figure 5c shows that the field required to induce threshold switching in both the SET and RESET states decreases as the thickness increases (F_TH_ = V_TH_/*u*_a_). This is due to avalanche multiplication, where a thicker OTS layer enhances the generation of carrier pairs at a given electric field. The redistribution of charged defects was limited to the near interface region. Therefore, the difference in the field to threshold switching between the LVS and HVS (ΔF_TH_) decreased with increasing film thickness from ΔF_TH_ ≈1.14 MV cm^−1^ in 6 nm‐thick to ΔF_TH_ ≈0.34 MV cm^−1^ in 40 nm‐thick. However, because the memory window was determined by multiplying the thickness by ΔF_TH_, it increased up to a 30 nm thickness and then decreased.

Finally, we examine the dependence on the device structure in the memory window.

The memory window of the mushroom‐type asymmetric cell (ΔV_TH_ ≈1.23 V) was ≈30% larger than that of the pillar‐type symmetric cell (ΔV_TH_ ≈0.94 V) (Figure S14, Supporting Information). The BE metals are different for the mushroom‐type (TiN) and pillar‐type (Carbon) devices, but we believe the main reason for the memory window difference lies in the device structure rather than the metal type. The measured work functions of TiN and metallic carbon are 4.8 and 4.6 V, respectively, showing no significant variation. On the other hand, for mushroom‐type cells, it has been reported that the memory window of GeSe materials can vary from 1 to 4 V depending on the BE diameter.^[^ ^13^ ^]^ However, TEM/EDS analysis confirmed that the compositional change after SET/RESET operation was minimal in both structures (within the EDS error range), thus we exclude the effect of atomic segregation/clustering (Figure S15, Supporting Information). As an alternative explanation, we attribute the enhanced memory window to asymmetric Joule heating in mushroom‐type cells, which increases the diffusivity of charged defects near the BE compared to the TE. The diffusivity of ionized atoms follows the Arrhenius law, *D* = *D*_0_exp(−*E*_a_/kT) and is directly proportional to temperature, where *E*_a_ is activation energy for diffusion and *D*_0_ is the pre‐exponential factor. This temperature‐driven asymmetric diffusivity amplifies the impact of writing pulse polarity, leading to the asymmetric defect distribution. Although the migration of these defects under the writing pulse affects carrier transport in subsequent read operations, it is difficult to observe compositional changes due to their low concentration in amorphous material (≈10^17^ cm^−3^). A better understanding of the increased memory window observed in asymmetric cells could greatly assist in optimizing the device structure. Further investigation is required to fully verify the effect of asymmetric heat generation and provide a more accurate physical interpretation.

## 6 Conclusion

This study experimentally and theoretically elucidated the mechanism behind the polarity‐dependent V_TH_ shift in OTS selectors based on amorphous chalcogenides. Notably, threshold switching in the same or opposite direction exhibited electrical characteristics corresponding to two distinct PF conduction models combined with avalanche multiplication. This phenomenon can be explained as follows: In the initial state, the first firing process generates a deep trap state as a carrier emission site for PF transport in the read operation. Subsequently, applying writing pulses in either the same or opposite direction creates varying gradients of the defect states, attributed to the high electric field near the electrodes. Threshold switching occurs when a large concentration of high‐mobility secondary carriers reaches a threshold current, I_TH_. In the pre‐switching region, the difference in the critical voltage required to reach the I_TH_ arises owing to the varying PF potential barrier lowering for carrier emission, becoming the memory window, ΔV_TH_. We proposed that the double‐ionized dimers are the microscopic origin of the polarity effect, as confirmed by DLTS spectrum and DFT simulations. In addition, we systematically investigated the effect of chemical compositions, film thickness, and device structure on the device performance. These findings accelerate the optimization and innovation of material systems. In phase‐change memory, significant improvements in device performance have been achieved by alloy design^[^ ^33^ ^]^ or by implementing new structures that can alternate the switching mechanism.^[^ ^34^, ^35^ ^]^ In future work, these strategies should be conducted to improve the trade‐off between device reliability and memory window in SOM applications. Furthermore, 3DXP memory is advantageous for high‐density integration of cells due to its stacking architecture, making it highly suitable for implementing high‐density memory application. However, as memory density increases, there are limitations in the peripheral area required for operation. To address this issue, recent studies have proposed vertical SSM, which modifies conventional 3DXP into a vertical structure that utilizes a common bit‐line. The theoretical interpretations and analyses of the SOM operation mechanism presented in this study lay the foundation for the performance, composition, and structural optimization of a new class of emerging memory and provide opportunities to meet the memory demands of the AI era with a low‐cost memory solution.

## 7 Experimental Section

### Device Fabrication and Characterization

A set of devices with OTS material was fabricated to measure the experimental parameters. Two device structures, mushroom‐ and pillar‐shaped cells, were investigated (Figure S16, Supporting Information). The device comprised an OTS film sandwiched between the top electrode (TE) and the bottom electrode (BE). In a mushroom‐type cell, the BE (TiN) was etched into a pillar with critical dimension (CD) defined down to 8 nm. A 6– 40 nm thick OTS film, along with TE metal, was deposited on top of BE, resulting in a mushroom‐type cell structure. A unique combinatorial sputtering system (CANON‐Anelva) was used for high‐throughput thin‐film material screening. This system can deposit 41 different compositions of thin‐film materials on a single wafer. The composition of the multicomponent chalcogenides was controlled by co‐sputtering with four tilted sputtering guns. The chemical composition of the co‐sputtered thin film was analyzed through inductively coupled plasma atomic emission spectroscopy (ICP‐AES) and X‐ray fluorescence spectrometry. In a pillar cell, the entire BE(carbon)/OTS/TE(carbon) stack was etched into a pillar with CD defined down to 16 nm. The pillar‐type device faced significant technological challenges, particularly in preserving the chalcogenide composition on the cell sidewalls during the complex etching steps. Unless otherwise noted, the mushroom‐type cell and Ge_20_As_30_Se_50_ composition was used for most electrical measurements.

Direct current (DC) measurements were performed using the source measurement unit (SMU) of the KEYSIGHT PXIe M9005A for the SOM device characterization. The pulse generator unit (PGU) of the KEYSIGHT 81150A, 81160A, along with the current waveform analyzer of the CX3324, was used for pulse measurements. V_TH_ and I_off_ were evaluated through DC *I–V* sweeps, and pulse measurements were used to assess the V_TH_ drift and memory window. The V_TH_ in the *I–V* curve was determined as the voltage value reaching the threshold current, while the I_off_ was calculated as the current value at the 0.6 * V_TH_ point. The V_TH_ drift was evaluated based on the V_TH_ shift depending on the delay time variable of the Write to Read pulse, ranging from 1 µs to 10 s (Figure S17, Supporting Information). The memory window was evaluated by measuring the change in V_TH_ based on the height and polarity of the Write pulse.

### Defect Analysis

An asymmetric OTS device was fabricated for defect analysis. The BE(W)/OTS(32 nm)/TE(Carbon) stack was used for the DLTS. Our ultraviolet photoelectron spectroscopy (UPS) analysis measured the work functions of metals as φ(Carbon) = 4.8 V and φ(W) = 4.55 V. The metallic carbon layer thickness was 10 nm as the TE. The bottom contacts were made in series of different diameters (0.5, 1.0, and 1.5 um). DLTS measurements were performed using a PhysTech FT 1030 DLTS system. The capacitance was measured using a modified Boonton 72B capacitance meter with a 1 MHz capacitance meter. Temperature scans were made between 80 and 400 K at a heating rate of 2 K min^−1^. The samples were placed in a liquid nitrogen cryostat. The pulse height, filling pulse width, and pulse period width were 0.5 V, 10 ms, and 10 ms, respectively. The activation energy and concentration of traps were calculated using an Arrhenius plot. In amorphous chalcogenides, the Fermi level was pinned to the middle of the bandgap owing to the high concentration of localized defect states near the middle of the bandgap. This resulted in a depletion layer confined to the vicinity of the metal–semiconductor interface (a few nanometers). However, the depletion layer was confirmed to extend deeper depending on the DLTS bias conditions. Furthermore, the DLTS signal in the initial state of the device was not measured owing to a low capacitance transient attributed to carrier emission. Thus, the near‐interface trap was negligible before the first firing. In the proposed system, a negative DLTS signal indicates that all the visible peaks correspond to the hole carrier traps with the energy levels measured from the VBM.

### Photonic *I–V* Measurement

The optical pumping method was used to analyze the trap profile through carrier excitation from the defect state in the wavelength range of 440–980 nm (Figure S1, Supporting Information). A supercontinuum laser of NKT Photonics FIANIUM was used as the optical pumping source. The measured photo current values were used to calculate the photo‐carrier (n_ph_) as a function of wavelength (λ) through the drift conduction equation. Subsequently, the relationship between photon energy (E_ph_) as a function of λ and the chain rule was used to extract the density of state (DOS).

### TEM/EDS Measurement

A focused ion beam (FIB) was used to prepare samples for TEM observation, which is a specific technique for site‐oriented milling of a sample typically using a Gallium ions beam focused down to a few nm. An investigation was conducted was over the microstructures and the composition of the GeAsSe materials using the Titan3 60–300 S/TEM, equipped with field emission electron gun (X‐FEG), a Cs‐double (image/probe) corrector and an electron gun monochromator, energy‐dispersive x‐ray spectroscopy (EDS) and electron energy loss spectroscopy (EELS).

### DFT Simulations

The density‐functional calculations were performed using the generalized gradient approximation (GGA) for the exchange‐correlation and projector augmented‐wave potentials, as implemented in the VASP code.^[^ ^36^ ^]^ A plane‐wave basis set was used to expand the wave functions with an energy cutoff of 500 eV, and a k‐point set was generated using a 2 × 2 × 2 gamma‐centered mesh for Brillouin zone integration. The ionic coordinates were fully optimized until the residual forces were less than 0.04 eV Å^−1^. In addition, hybrid functional calculations of Heyd, Scuseria, and Ernzerhof (HSE) were performed to obtain reliable bandgaps and defect levels.^[^ ^37^ ^]^ Diverse compositions of amorphous models for chalcogenides were created through melt‐and‐quench ab initio molecular dynamics (AIMD) simulations. To avoid the stochastic effect in amorphous, ten different amorphous models were considered in each Ge_x_As_y_Se_100‐x‐y_ composition, where the species fraction was x = 10%, 20%, 30%, 40%, and 50% and y = 10%, 20%, 30%, 40%, and 50%, and the minimum Se concentration was 40%. Each supercell contained 200 atoms. The time step used was 3 fs, and the cutoff energy of the plane‐wave basis was set to 400 eV in the AIMD simulations. These models were further relaxed at 0 K to calculate the electronic structures.

Twenty different configurations of Se*_i_* and *V*_Se_ defects were generated by inserting a guest Se atom in various interstitial and vacancy positions in *a*‐GAS. The formation energy of Se*_i_* and *V*_Se_ defects in the charge state *q* was calculated as a function of Fermi energy *E*_F_, expressed as follows:

(1) $$E^{f}\;\left[X_{q}\right]=E_{tot}\;\left[X_{q}\right]-E_{tot}^{0}-\sum_{i}n_{i}^{Se}\mu_{Se}+q\left(E_{VBM}^{0}+E_{F}\right)$$

where *E_tot_* [*X_q_*] and $E_{tot}^{0}$ are the total energies of the supercell with and without defects, respectively, $E_{VBM}^{0}$ is the energy of the VBM from the DFT calculations, and $n_{i}^{Se}$ is the number of Se atoms added or removed as a defect. The Se chemical potential, µ_Se_, was set under Se‐poor and Se‐rich conditions. In the Se‐poor condition, µ_Se_ was calculated using the total energy of the bulk Se phase (*E*_Se_) and the formation enthalpy of GeSe (Δ*H*_GeSe_), expressed as µ_Se_ = *E*_Se_  + Δ*H*_GeSe_. Conversely, under Se‐rich conditions, µ_Se_ was determined from the bulk phase and estimated to be ‐4.45 eV based on the HSE calculations. Because *a*‐GAS has tail states owing to structural disorder and defects, the logarithm of the inverse participation ratio was calculated to determine the band‐edge states.^[^ ^38^ ^]^

## Conflict of Interest

The authors declare no conflict of interest.

## Author Contributions

H.‐J.S. and M.C. contributed equally to this work. C.S.L. and K.Y. planned and supervised the study. H‐.J.S. conducted the DFT calculations, material modeling, and manuscript writing. M.C. and S.H. conducted most of the device characterizations and defect analyses. Z.W. and H.C. supported device fabrication. B.K., J.‐B.P., W.Y., Y.K., Y.P., and Y.H. supported the combinatorial sputter deposition, TEM, and ICP‐AES analysis, respectively. All of the authors contributed to the data analysis.

## Supporting information



Supporting Information

## Data Availability

The data that support the findings of this study are available on request from the corresponding author. The data are not publicly available due to privacy or ethical restrictions.
