# Supplementary material for: Microscopic Origin of Polarity‐Dependent VTH Shift in Amorphous Chalcogenides for 3D Self‐Selecting Memory
Source: Adv Sci (Weinh). 2024 Oct 9;11(44):2408028. doi: 10.1002/advs.202408028 (PMC11600224; doi:10.1002/advs.202408028)
Supplement: Supplementary file 1 — Supporting Information [file ADVS-11-2408028-s001.docx]

Supporting Information

**Microscopic origin of polarity-dependent V_TH_ shift in amorphous chalcogenides for 3D self-selecting memory**

Ha-Jun Sung*^1^, Minwoo Choi*^1^, Wu Zhe^2^, Hwasung Chae^2^, Sung Heo^3^, Youngjae Kang^1^, Bonwon Koo^1^, Jongbong Park^1^, Wooyoung Yang^1^, Yongyoung Park^1^, Yongnam Ham^1^, Kiyeon Yang^1^, and Chang Seung Lee^1^

**Affiliations**

^1^Thin Film Technical Unit, Samsung Advanced Institute of Technology, Samsung Electronics, Suwon-si, South Korea

^2^Advanced Process Development Team, Semiconductor R&D Center, Samsung Electronics, Hwaseong-si, South Korea

^3^Analytical Engineering Group, Samsung Advanced Institute of Technology, Samsung Electronics, Suwon-si, South Korea

Corresponding author. Email: kiyeon.yang@samsung.com (K.Y.), cielolee@samsung.com (C.S.L.)

*These authors contributed equally

**Section 1.** Carrier capture process in dimer type defects.

**Section 2.** Read operation scheme in SOM device.

Figure S1. Photonic I-V method to extract density of states.

Figure S2. The extracted impact ionization (II) coefficient α.

Figure S3. The total DOS and PDOS onto the intimate valance alternation pair atoms in amorphous Se.

Figure S4. Calculated total energies as a function of the Se dimer and cation dimer distance.

Figure S5. The total DOS and PDOS onto Se dimer and cation dimer in neural and charged states of amorphous Ge-As-Se.

Figure S6. The average formation energies of the *V*_Se_ and Se*_i_* defects.

Figure S7. Color map of measured ΔV_TH_ and V_TH_ drift.

Figure S8. Ab-initio molecular dynamics (AIMD) simulations.

Figure S9. Operating scheme with write pulse in positive and negative read.

Figure S10. Endurance performance of SSM device under cyclic RESET/SET writing pulse.

Figure S11. The comparison of memory window obtained from V-V curve of mushroom- and pillar-type cell.

Figure S12. Repeated operation speed measurement as a function of write pulse duration.

Figure S13. Operation yield and uniformity of OTS devices achieved using combinatorial methodologies.

Figure S14. Current measured versus pulse duration.

Figure S15. TEM/EDS data of mushroom and pillar-type devices.

Figure S16. Schematic of Ge-As-Se SOM device in mushroom- and pillar-type device.

Figure S17. Typical DC characteristics of TE/carbon/OTS/TE devices.

Table S1. Comparison of electrical characteristics of a variety composition of SOM devices.

**Section 1.** Carrier capture process in dimer type defects.

The concentration of dimer defects was estimated to be around 10^20^~10^21^ cm^-3^ based on the creation energy, but it is important to note that this estimation is based on the assumption of a neutral dimer defect in the as-deposited amorphous structures. DLTS analysis measures the transient of traps where carriers are captured, meaning that only ionized defects are detected. The concentration of E2 and H2 defects is measured as 1.3×10^17^ and 1.0×10^17^ cm^-3^, respectively. For a neutral dimer to be ionized, it must capture carrier by overcoming an activation barrier, resulting in a broken dimer state. Here, we plot the calculated total energies as a function of the dimer distance in **Figure S4**. The capture barrier can be estimated from the configuration diagram in which the energies of the dimer defects in doubly charged states. The energy barrier in the Se and cation dimer to broken state transition is found to be 0.41 and 0.27 eV, respectively. Thus, the concentration of ionized dimer defects is quietly reduced compared to the neutral states. In the neutral state, the broken dimer is found to be meta-stable and prefers to be on the contrary to the carrier-injected case. The broken dimer state is higher in energy by 1.12 eV and 0.98 eV than the Se and cation dimer state, respectively. That is the reason why the broken dimer defects are not preferentially formed in the as-deposited amorphous structures.

**Section 2.** Read operation scheme in SOM device.

For positive read scheme, performing a SET write (t_width_ = 100 ns, V_Write_ > $V_{\mathrm{HVS}}^{P}$) will place the device in an LVS state. The next read pulse, V_Read_ = ($V_{\mathrm{LVS}}^{P}$ + $V_{\mathrm{HVS}}^{P}$)/2 will turn on the device, indicating a logic "1" state (**Figure S9a**). If a second identical polarized read operation is performed, the "1" state is maintained, and the I-V curve remains almost identical, providing no additional information (**Figure S9b**). For a RESET write, applying an opposite polarity pulse greater than V_HVS_ will place the device in an HVS state. Even when the next read pulse is applied, the device does not turn on, indicating a logic "0" state (**Figure S9c**). Therefore, the read operation of the SOM device is nondestructive under this pulse scheme.


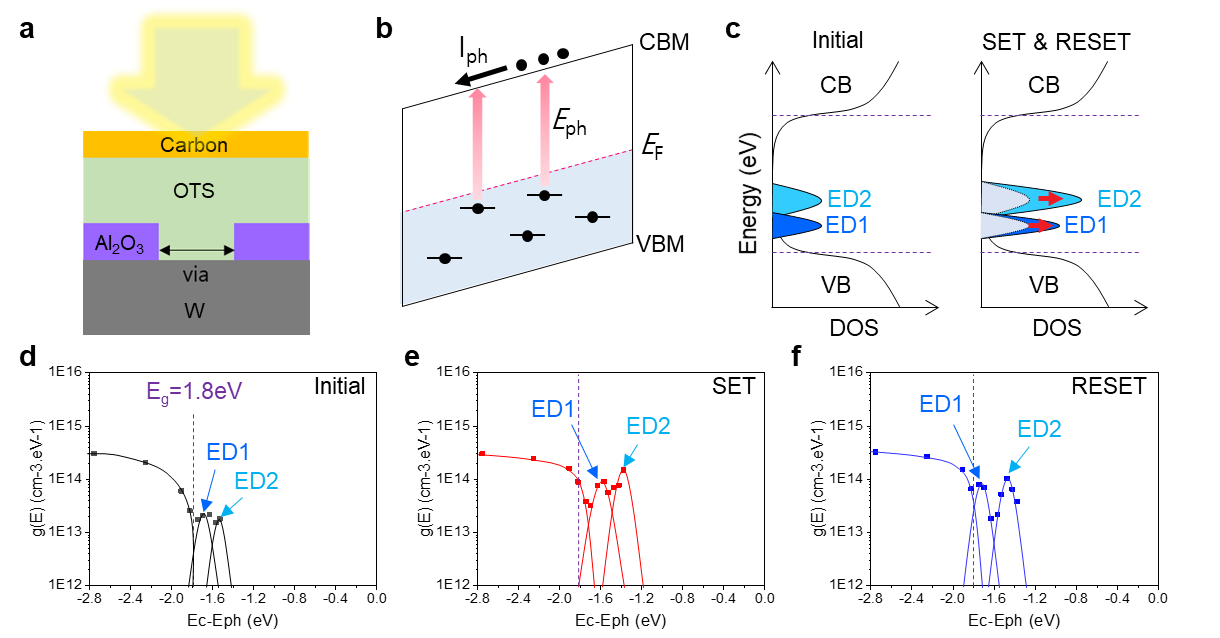


**Figure S1 Photonic I-V method to extract density of states.** Schematic diagram of **a** device structure for photonic I-V measurement. The laser is irradiated through thin (10 nm) carbon metal layer. **b** Process of photo carrier generation from trap states. **c** Band diagram from experimentally obtained DOS of Ge-As-Se OTS. Obviously, the concentration of ED1 and ED2 state increase after firing process. Extracted DOS profile by photonic I-V measurement for **d** initial, **e** SET, and **f** RESET states in our device. The band gap is obtained from the optical measurement via ellipsometry.


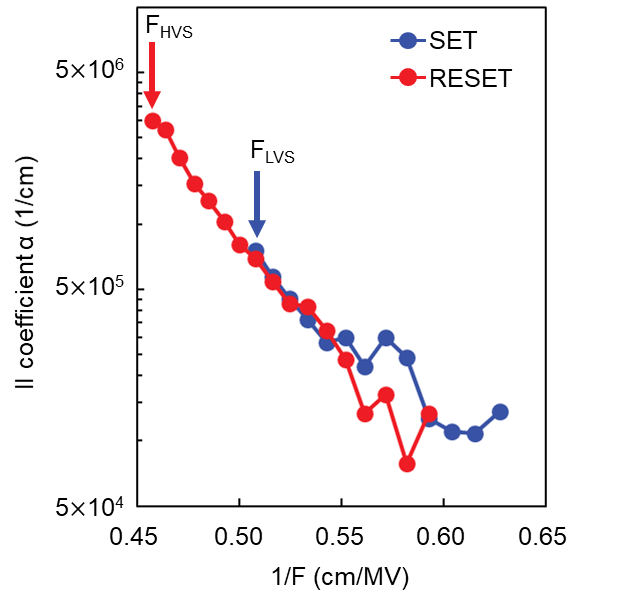


**Figure S2.** The extracted impact ionization (II) coefficient α from measured M factor.


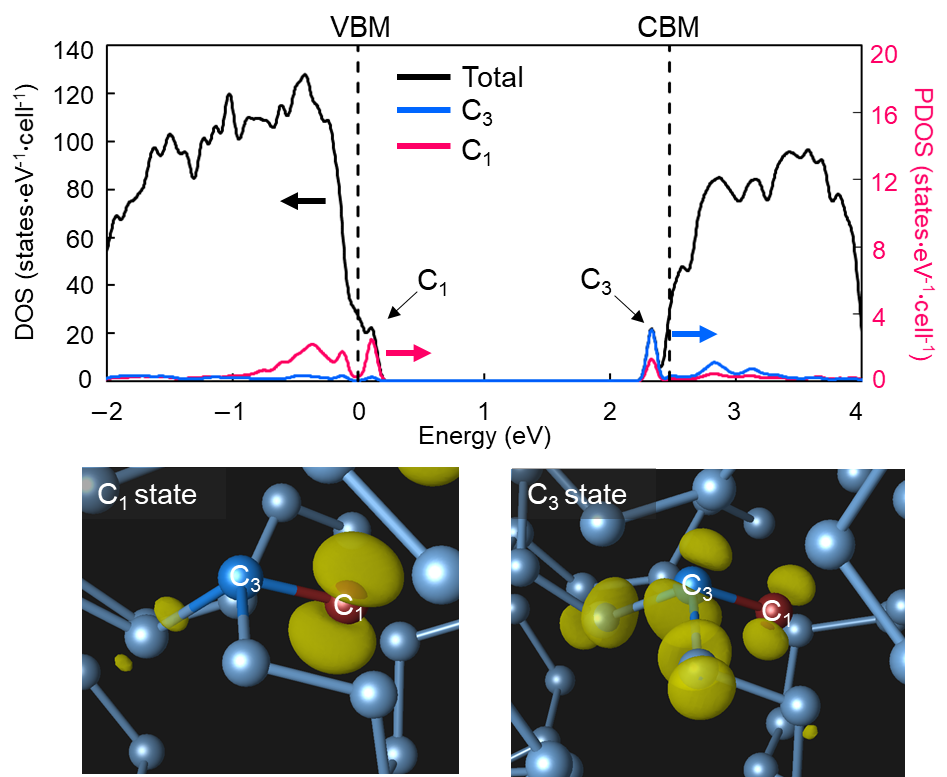


**Figure S3.** The total DOS and PDOS onto the intimate valance alternation pair atoms in amorphous Se. The C_1_ and C_3_ center atoms denote the red and blue spheres, respectively. The VBM and CBM state are determined by using the LIPR values. The extracted band gap is 2.4 eV. Isosurfaces of the charge densities for the C_1_ and C_3_ state.


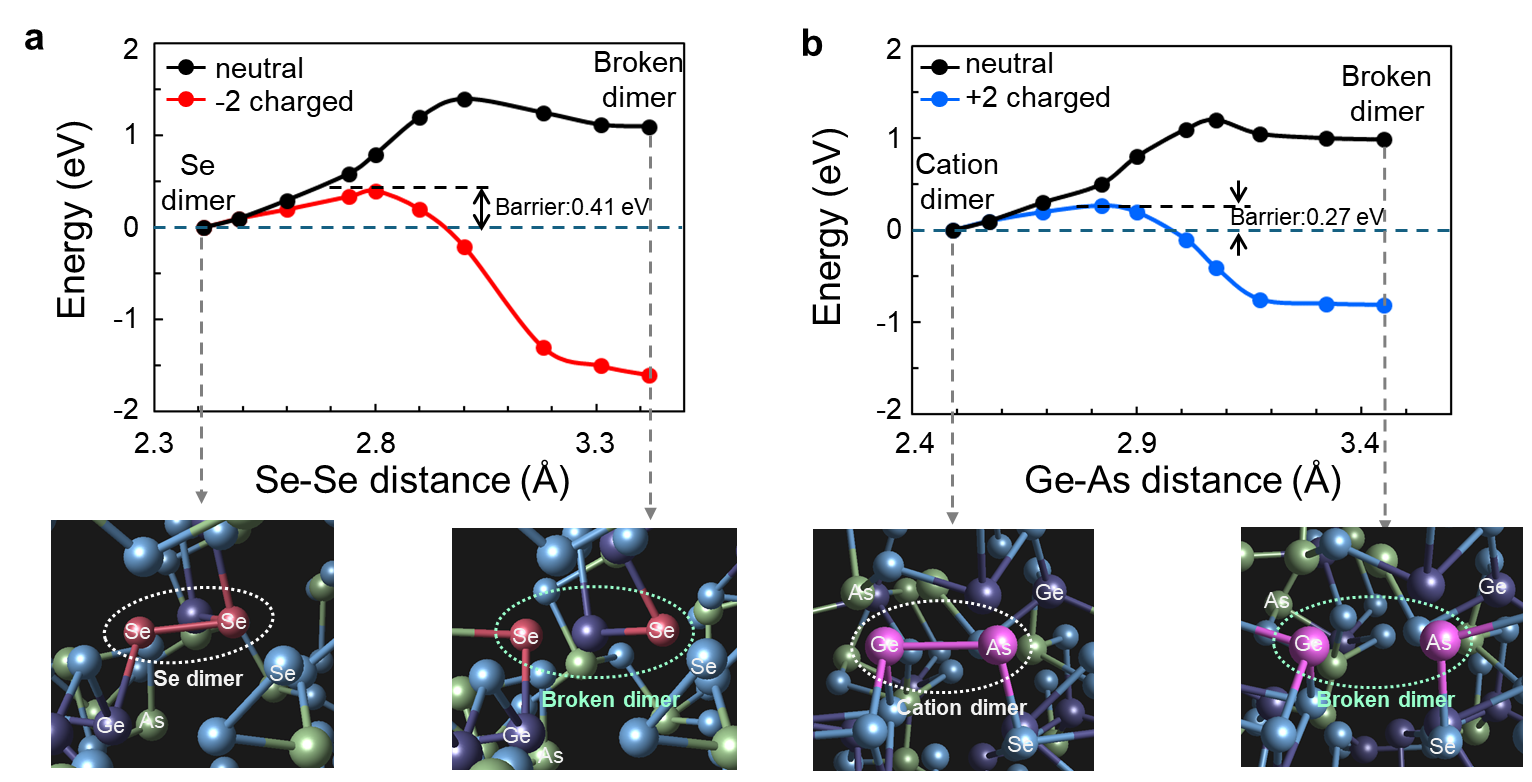


**Figure S4.** Calculated total energies as a function of the **a** Se dimer and **b** cation dimer distance, with respect to the dimer state energy in HSE functional calculations.


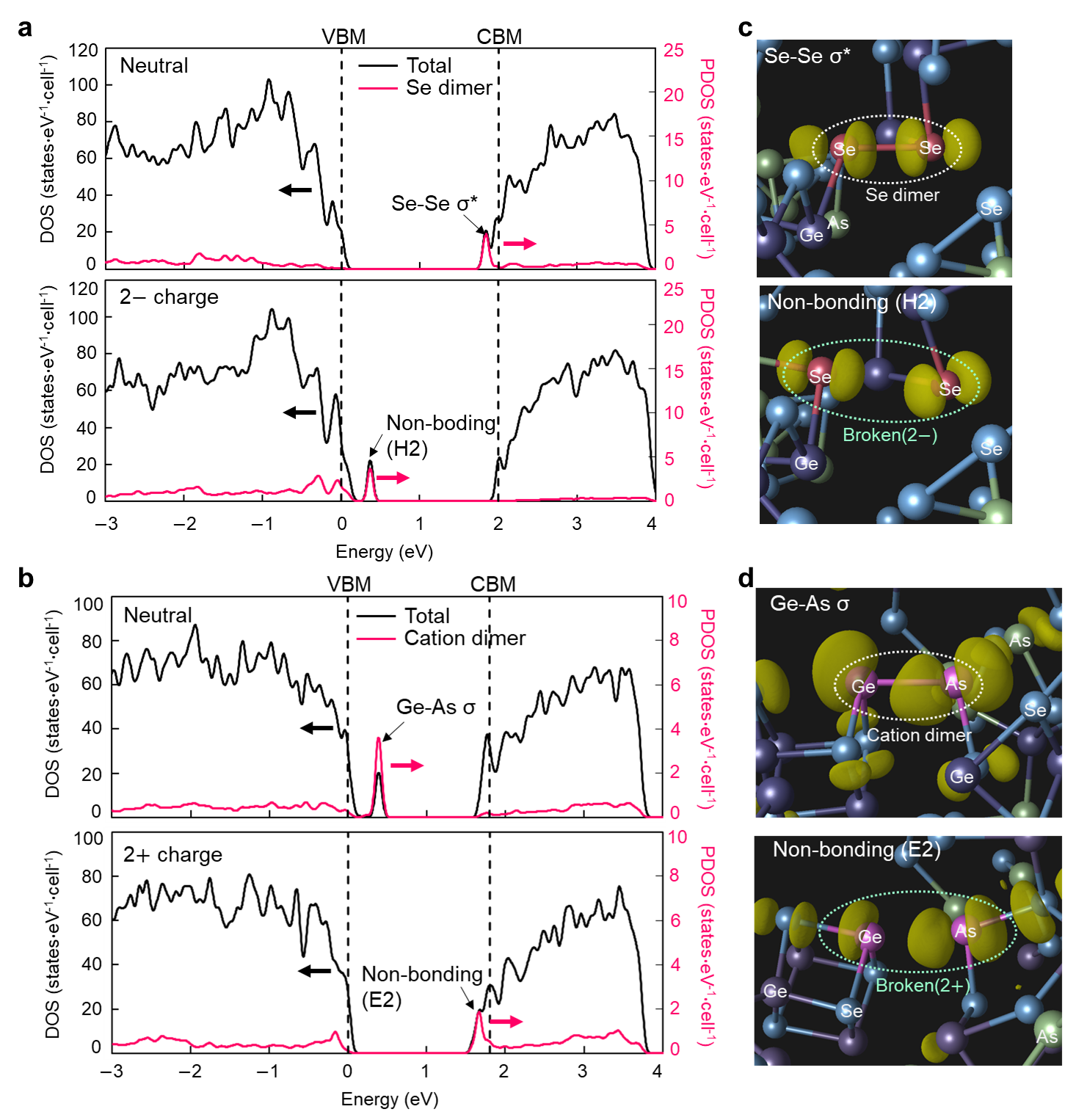


**Figure S5.** The total DOS and PDOS onto **a** Se dimer atoms in the neutral (upper) and 2- charge states (lower) of Ge_20_As_20_Se_60_ and **b** cation dimer atoms in the neutral (upper) and 2+ charge states (lower) of Ge_30_As_20_Se_50_, with the VBM set to zero. The VBM and CBM state are determined by using the logarithm of the inverse participation ratio (LIPR) values. The extracted band gap is 2.0 and 1.8 eV for amorphous Ge_20_As_30_Se_50_ and Ge_30_As_20_Se_50_, respectively. Isosurfaces of the charge densities for the **c** Se dimer and **d** cation dimer. The calculated defect level is corresponding to the Figure 4 in main text.


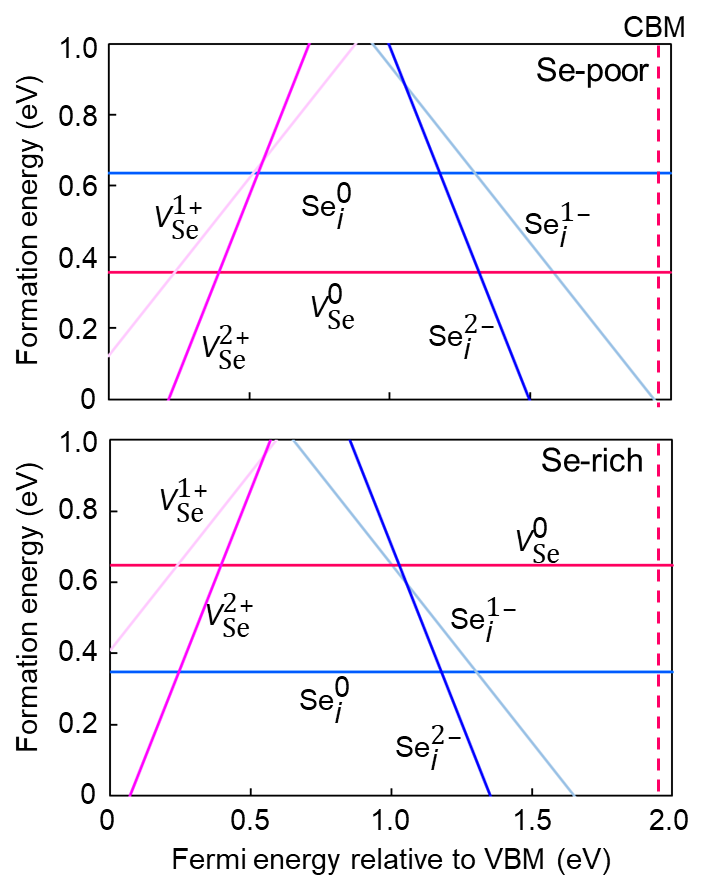


**Figure S6**. The average formation energies of the *V*_Se_ and Se*_i_* defects for amorphous Ge_20_As_20_Se_60_ as a function of Fermi energy relative to VBM under the Se-poor (top panel) and Se-rich (bottom panel) condition in the HSE functional calculations.


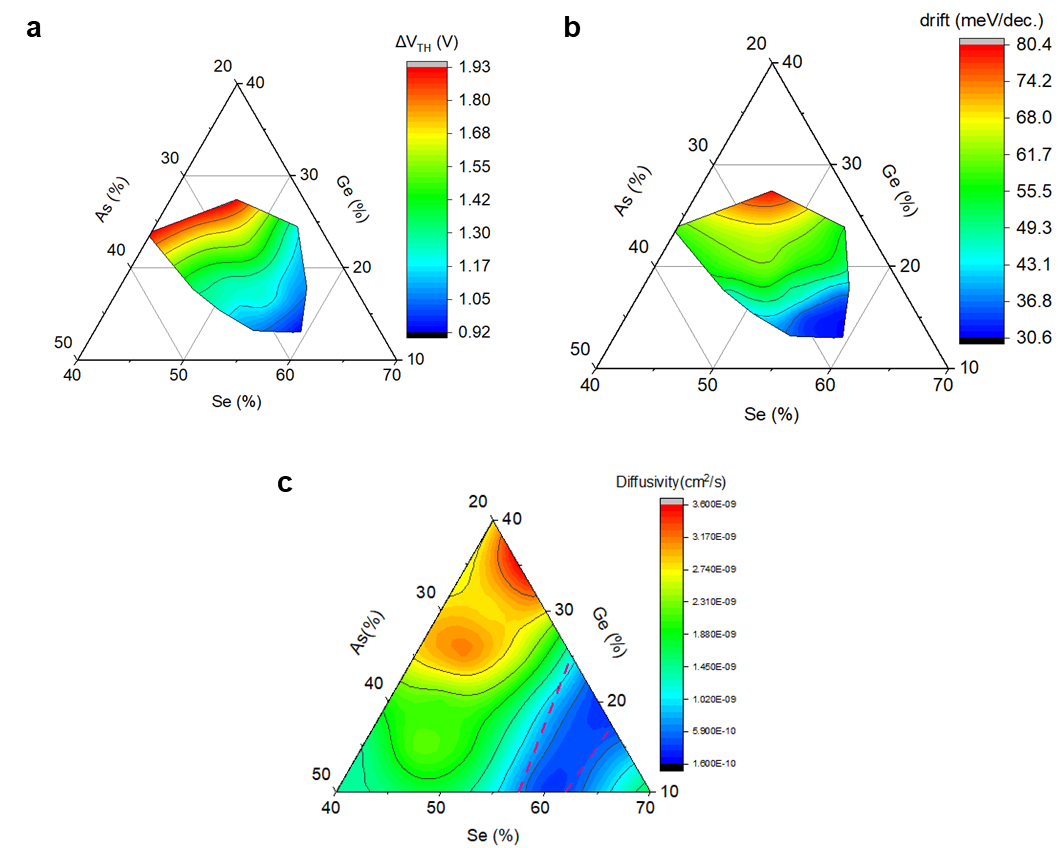


**Figure S7.** Color map of measured **a** memory window ΔV_TH_ and **b** V_TH_ drift for Ge-As-Se system.


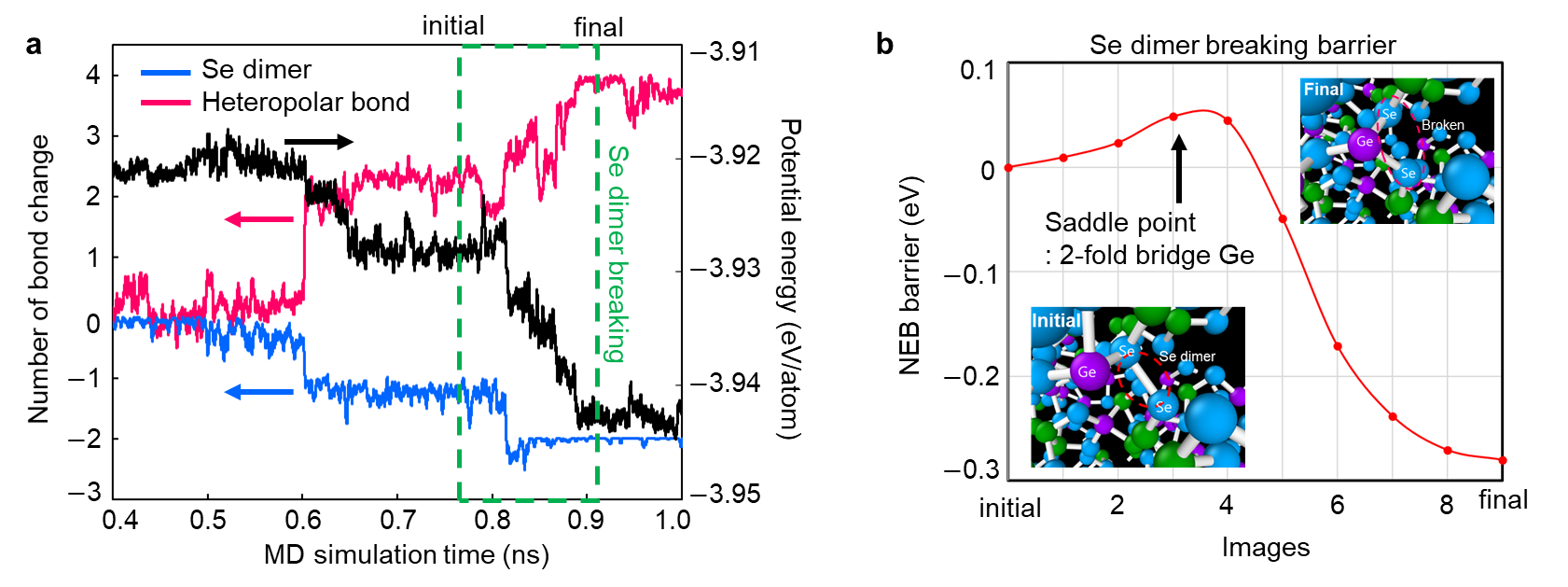


**Figure S8.** Ab-initio molecular dynamics (AIMD) simulations. **a** Change in bonding configuration from Se dimer to As-Se or Ge-Se heteropolar bond as a function of time. Green dashed box denote the structural relaxation process of Se dimer broken with amorphous stabilization during AIMD simulation. **b** Relaxation path from NEB calculations showing the energy barrier of 48 meV for the Se dimer broken. The initial and final structure is selected from AIMD.


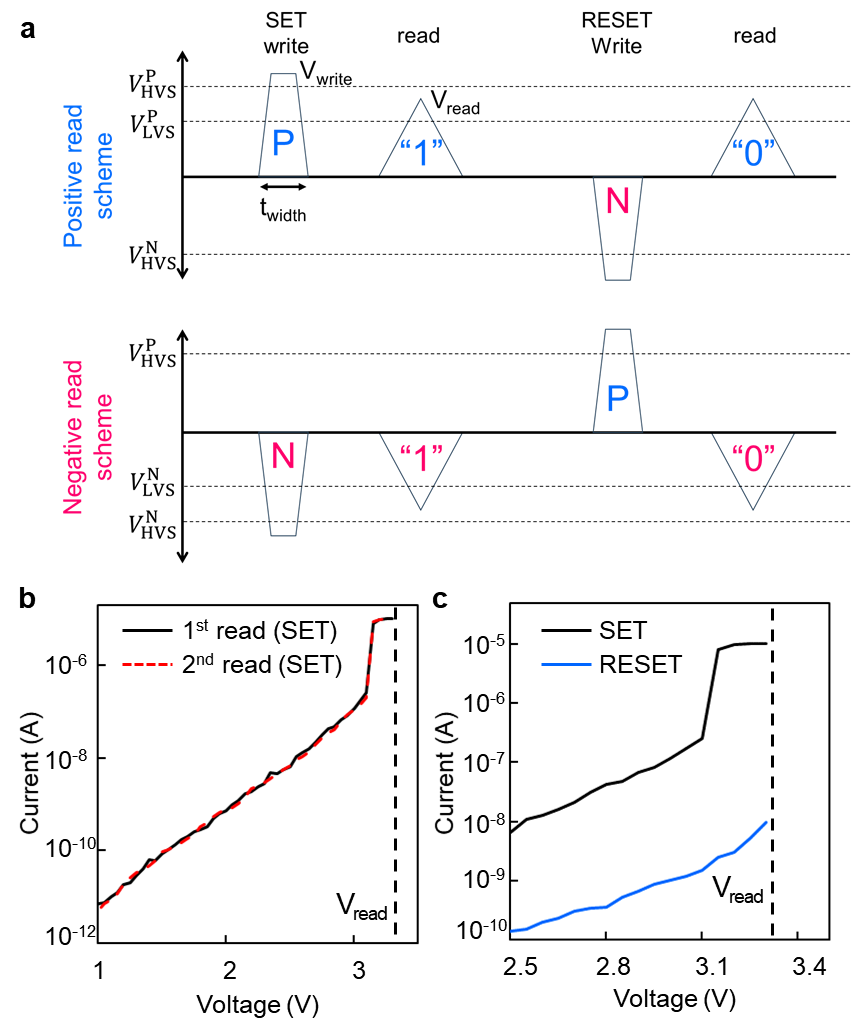


**Figure S9** **a** Operating scheme with write pulse in positive and negative read. **b** I-V characteristics with double read operation after SET write. **c** Measured I-V curve for different write polarity (SET and RESET).


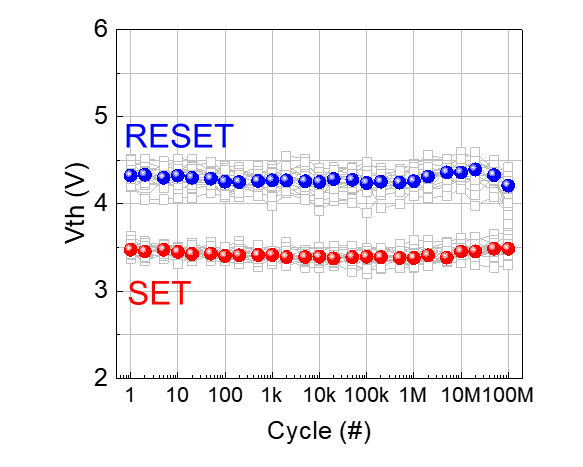


**Figure S10** Endurance performance of pilar-type device under cyclic RESET/SET writing pulse. To avoid the Se migration under repeated cycling stress, we used low Se composition (Se ~40%) material in endurance measurement.


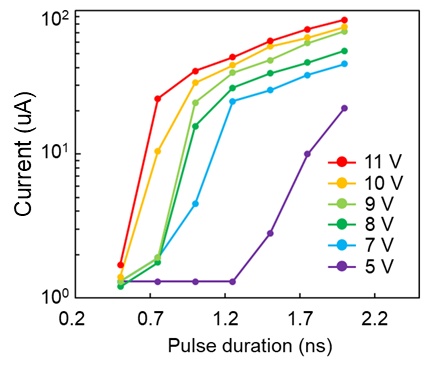


**Figure S11.** Current measured versus pulse duration. Our OTS device achieves stable threshold switching with the fast operation speed of 750 ps.


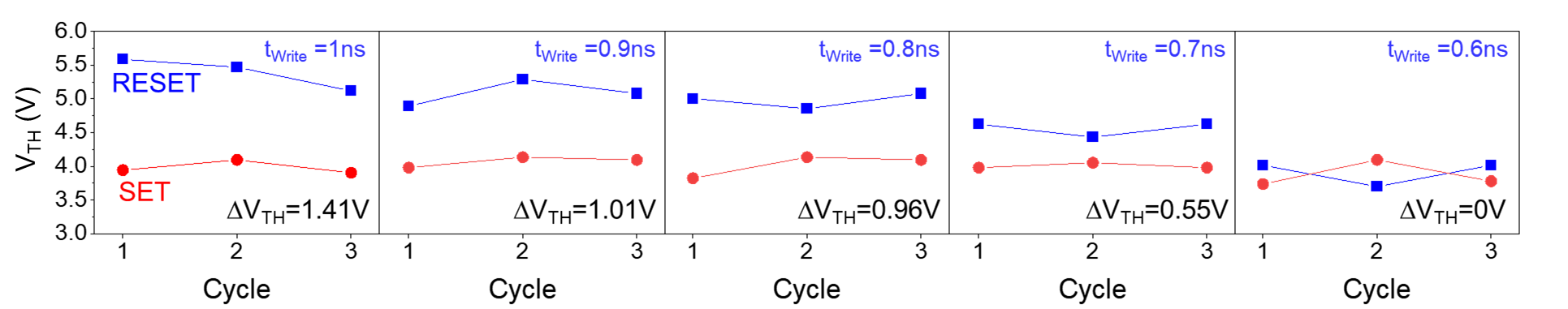


**Figure S12.** Repeated operation speed measurement as a function of write pulse duration time under 1ns.

**
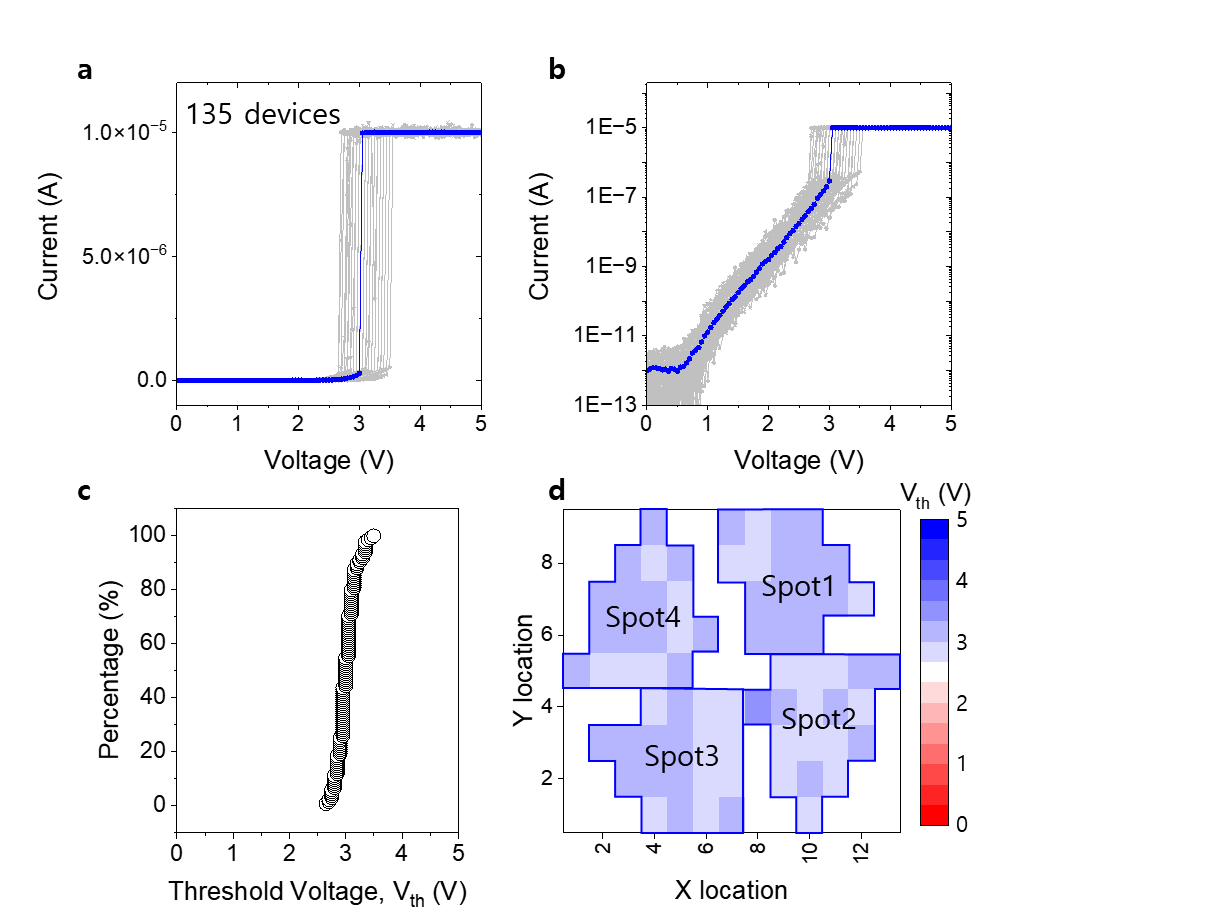
**

**Figure S13.** Operation yield and uniformity of OTS devices achieved using combinatorial methodologies. Plots of **a**-**b** the DC I-V characteristics, **c** V_TH_ uniformity of 135 devices. **d** Wafer mapping result of device performance according to the X and Y locations.


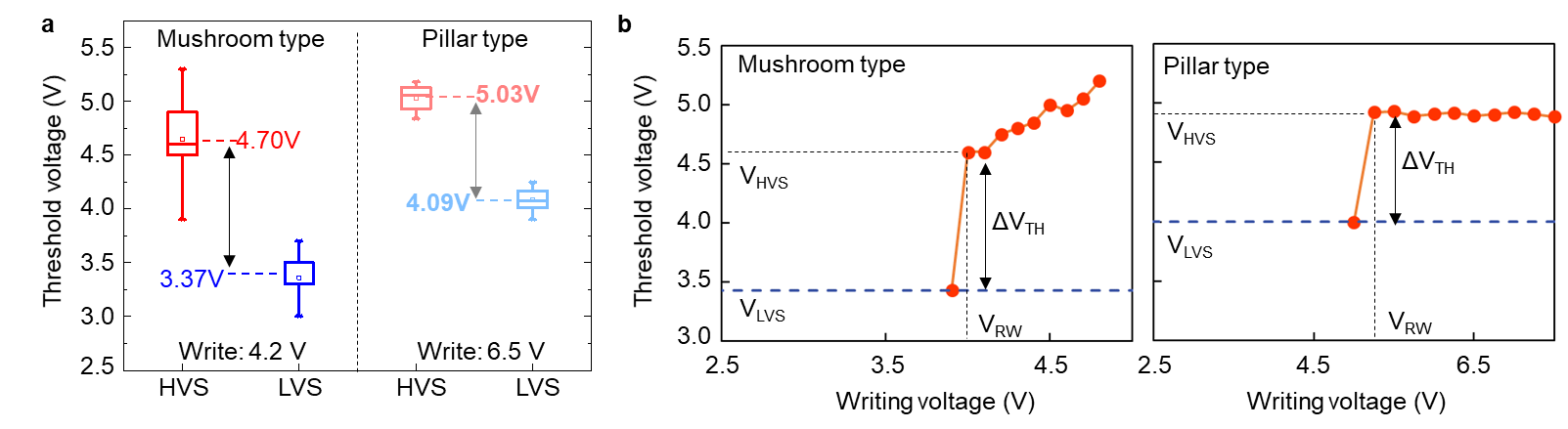


**Figure S14.** **a** The comparison of memory window obtained from V-V curve of mushroom- and pillar-type cell. **b** measured V-V curve in two device structures. (20 cells)


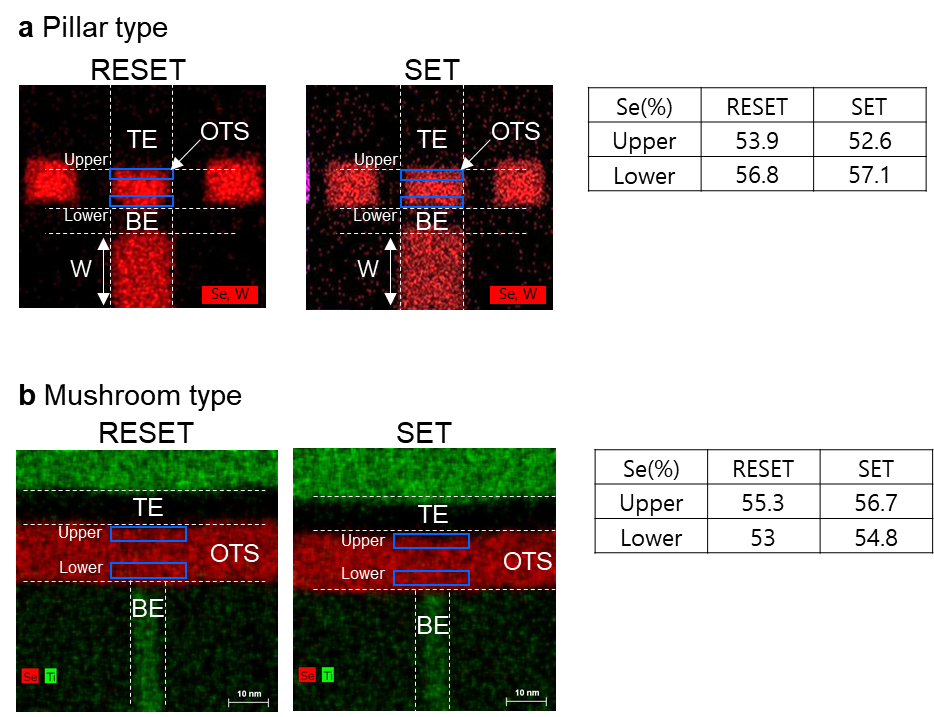


**Figure S15.** Energy-dispersive X-ray spectroscopy (EDS) map of Se elements in **a** pillar-type device and **b** mushroom-type device programmed with SET and RESET pulses. The red block on the sides of OTS is dummy cell of OTS and it shows Se component. The averaged concentration of Se in the upper and lower regions denote at the right table. Upper and lower regions are denoted as the blue box in the TEM/EDS map.


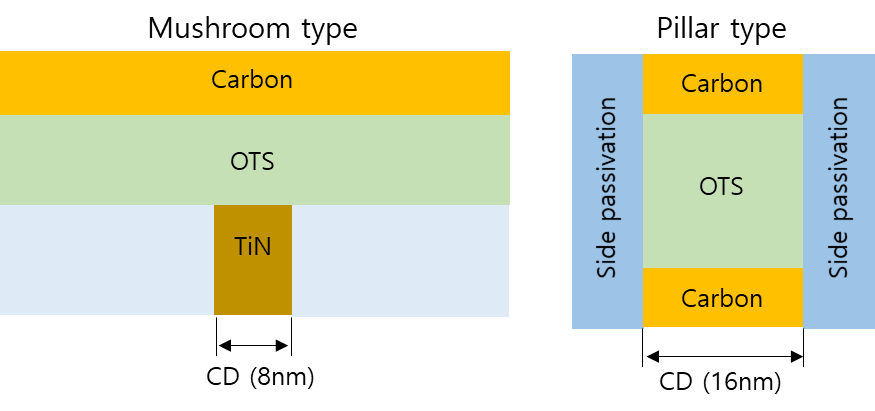


**Figure S16.** Schematic of Ge-As-Se SOM device in mushroom- and pillar-type device.


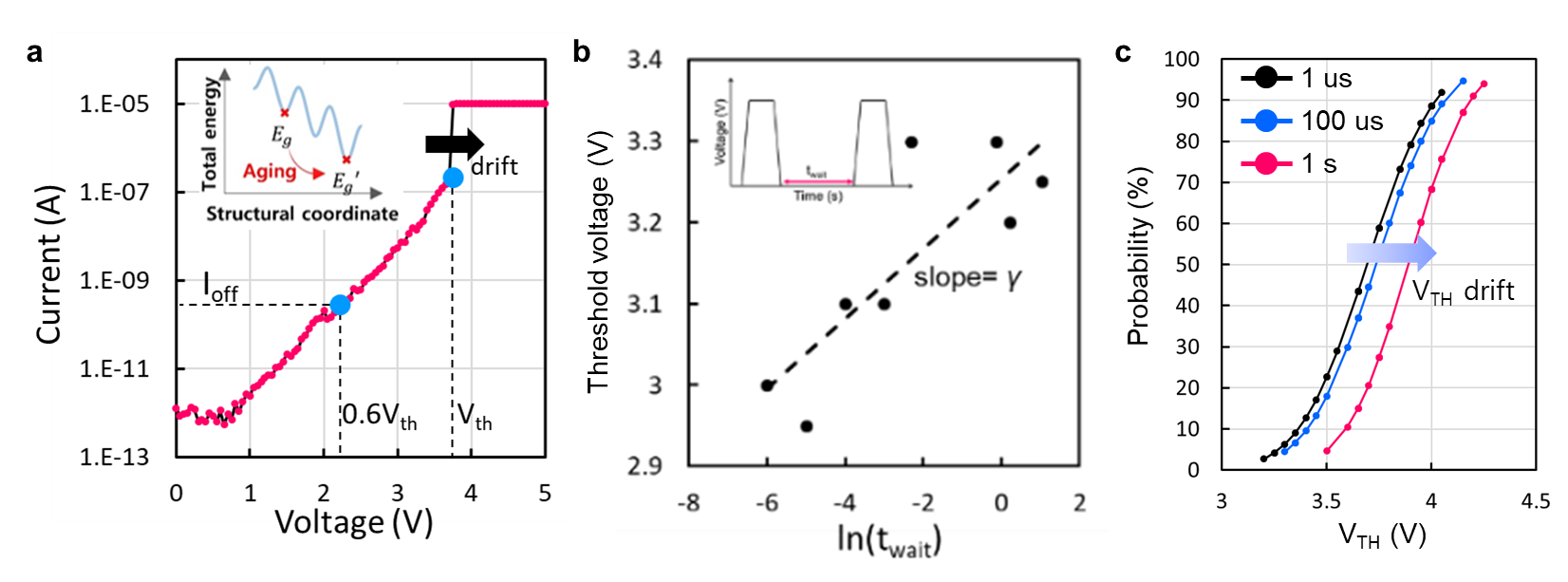


**Figure S17.** **a** Typical DC characteristics of TE/carbon/OTS/TE devices. The V_TH_ increase with the time evolution, called drift phenomena. **b** Drift of the OTS device is characterized by an increase in V_TH_ when t_WAIT_ between the pulses increases. Drift coefficient (γ) can be obtained by slope of linear regression in V_TH_ data with t_WAIT_. **c** Distribution of V_TH_ measured across 20 cells under different t_WAIT_ (1us, 100us, and 1s). We conducted the experiments at 85°C, which is higher than room temperature, to accelerate the aging and reliability tests of the device

**Table S1.** Comparison of electrical characteristics of a variety composition of SOM devices.

|  | Se (%) | V_TH_ (V) | I_off_ (nA) | Drift  (mV/dec.) | ΔV_TH_ (V) |
| --- | --- | --- | --- | --- | --- |
| d1 | 39 | 3.00 | 0.63 | 59 | 1.94 |
| d2 | 44 | 2.75 | 1.44 | 55 | 1.36 |
| d3 | 45 | 3.05 | 0.45 | 65 | 1.64 |
| d4 | 45 | 3.35 | 0.32 | 80 | 1.93 |
| d5 | 46 | 2.90 | 0.97 | 42 | 1.27 |
| d6 | 48 | 2.95 | 1.02 | 60 | 1.50 |
| d7 | 48 | 3.30 | 1.67 | 48 | 1.30 |
| d8 | 50 | 2.95 | 0.48 | 60 | 1.35 |
| d9 | 50 | 2.80 | 0.87 | 48 | 1.25 |
| d10 | 50 | 3.20 | 0.66 | 58 | 1.22 |
| d11 | 50 | 3.43 | 2.26 | 43 | 1.23 |
| d12 | 50 | 3.08 | 0.83 | 52 | 1.26 |
| d13 | 51 | 2.80 | 0.88 | 52 | 1.20 |
| d14 | 51 | 2.85 | 0.95 | 55 | 1.02 |
| d15 | 51 | 3.00 | 0.80 | 42 | 1.15 |
| d16 | 51 | 2.90 | 1.19 | 46 | 1.13 |
| d17 | 52 | 2.70 | 1.49 | 37 | 1.06 |
| d18 | 52 | 3.20 | 0.43 | 49 | 1.23 |
| d19 | 52 | 3.10 | 0.44 | 52 | 1.28 |
| d20 | 53 | 3.00 | 0.80 | 39 | 1.15 |
| d21 | 53 | 3.05 | 0.61 | 45 | 1.27 |
| d22 | 54 | 3.15 | 0.79 | 43 | 1.22 |
| d23 | 54 | 3.05 | 0.61 | 54 | 1.13 |
| d24 | 54 | 3.00 | 0.60 | 33 | 1.15 |
| d25 | 54 | 2.95 | 0.70 | 33 | 1.07 |
| d26 | 55 | 2.85 | 0.95 | 41 | 1.10 |
| d27 | 55 | 3.35 | 1.61 | 34 | 1.08 |
| d28 | 56 | 3.05 | 0.45 | 37 | 1.05 |
| d29 | 57 | 2.65 | 2.00 | 31 | 0.92 |
| d30 | 57 | 2.80 | 1.11 | 33 | 0.96 |
